# Engineering yeast for high-level production of stilbenoid antioxidants

**DOI:** 10.1038/srep36827

**Published:** 2016-11-11

**Authors:** Mingji Li, Konstantin Schneider, Mette Kristensen, Irina Borodina, Jens Nielsen

**Affiliations:** 1 The Novo Nordisk Foundation Center for Biosustainability, Technical University of Denmark, DK-2970 Hørsholm, Denmark; 2 Department of Biology and Biological Engineering, Chalmers University of Technology, SE-41296 Gothenburg, Sweden; 3 The Novo Nordisk Foundation Center for Biosustainability, Chalmers University of Technology, SE-41296 Gothenburg, Sweden

## Abstract

Stilbenoids, including resveratrol and its methylated derivatives, are natural potent antioxidants, produced by some plants in trace amounts as defense compounds. Extraction of stilbenoids from natural sources is costly due to their low abundance and often limited availability of the plant. Here we engineered the yeast *Saccharomyces cerevisiae* for production of stilbenoids on a simple mineral medium typically used for industrial production. We applied a pull-push-block strain engineering strategy that included overexpression of the resveratrol biosynthesis pathway, optimization of the electron transfer to the cytochrome P450 monooxygenase, increase of the precursors supply, and decrease of the pathway intermediates degradation. Fed-batch fermentation of the final strain resulted in a final titer of 800 mg l^−1^ resveratrol, which is by far the highest titer reported to date for production of resveratrol from glucose. We further integrated heterologous methyltransferases into the resveratrol platform strain and hereby demonstrated for the first time *de novo* biosynthesis of pinostilbene and pterostilbene, which have better stability and uptake in the human body, from glucose.

Resveratrol (3,5,4′-trihydroxystilbene) is a natural plant defense compound with strong antioxidant activity. The therapeutical effects of resveratrol in humans are not documented in terms of mode of action and molecular target, but there are several reports on its efficacy for treatment of cardiovascular diseases^1^,^2^,^3^, cancer^4^,^5^ and aging^6^ in mice. This makes resveratrol a promising compound for applications as dietary supplement, functional food ingredient, cosmetics ingredient, and even as a therapeutic. Several derivatives of resveratrol have been created, where the methylated derivatives pinostilbene and pterostilbene showed better stability and uptake^7^. The market for resveratrol and its derivatives is expected to grow further in the future. Currently, resveratrol is predominantly extracted from Japanese knotweed *Polygonum cuspidatum*; however the process is dependent on the variable harvest, has low extraction yield and results in a low-purity product^8^. Production of resveratrol by microbial fermentation presents an alternative process circumventing the mentioned disadvantages of extraction from plants. Recombinant production of resveratrol was first shown in *S. cerevisiae* in 2003^9^. Several groups subsequently improved the production of resveratrol and its derivatives in yeast and *Escherichia coli*. However, all of these studies applied feeding of expensive precursors, such as *p*-coumaric acid^10^,^11^,^12^,^13^,^14^,^15^, tyrosine^15^,^16^,^17^ or phenylalanine^18^. The highest reported titer of resveratrol was 2.3 g l^−1^, when 2.5 g l^−1^ *p*-coumaric acid was fed to engineered *E. coli*^19^. We previously described the biosynthesis of resveratrol directly from glucose and ethanol, via the tyrosine pathway in yeast, which resulted in production of up to 531 mg l^−1^ resveratrol in fed-batch fermentation^20^. Here, we describe the development of a *S. cerevisiae* platform strain for production of resveratrol via phenylalanine pathway. The platform strain was obtained through extensive metabolic engineering of both the resveratrol pathway and pathways forming precursors for its biosynthesis, and it demonstrates clearly that for efficient production of plant chemicals by microbial fermentation it is necessary to combine pathway reconstruction with engineering of the endogenous metabolism^21^. We evaluated the platform strain for high-level production of resveratrol and also demonstrated its use for production of resveratrol derivatives.

## Results

### Reconstruction of resveratrol biosynthetic pathway in *S. cerevisiae*

**Figure 1:**
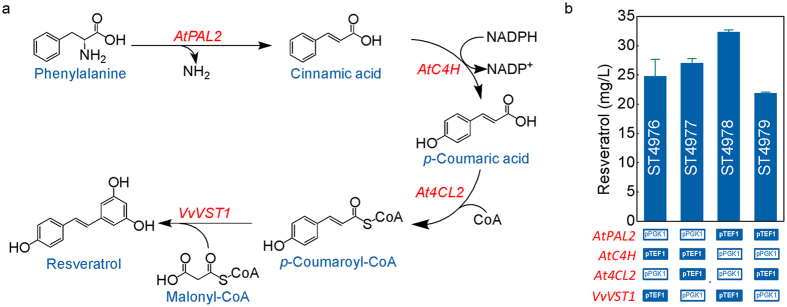
(**a**) The resveratrol biosynthetic pathway. *AtPAL2*, phenylalanine ammonia lyase from *A. thaliana*; *AtC4H*, cinnamic acid hydroxylase from *A. thaliana*; *At4CL2, p*-coumaryl-CoA ligase from *A. thaliana*; *VvVST1*, resveratrol synthase from *V. vinifera*. (**b**) Resveratrol production by engineered strains expressing the four biosynthetic genes from different promoters. The resveratrol concentration in the broth was measured after the cells were cultivated on mineral medium with 2% glucose and 5 mM phenylalanine for 72 hours in 96-deep-well plates. The displayed average values and standard deviations were calculated from biological triplicates.

The resveratrol biosynthesis pathway (Fig. 1a) was reconstructed in yeast by introducing phenylalanine ammonia lyase (*AtPAL2*), cinnamic acid hydroxylase (*AtC4H*), *p*-coumaryl-CoA ligase (*At4CL2*) from *Arabidopsis thaliana* and resveratrol synthase (*VvVST1*) from *Vitis vinifera*. Two strong constitute promoters, pTEF1 and pPGK1, were employed to control gene expression in different combinations. Engineered cells were cultivated on mineral medium supplemented with 5 mM phenylalanine for 72 hours to obtain 20–33 mg l^−1^ of resveratrol (Fig. 1b). No by-products (cinnamic acid or *p*-coumaric acid) were detected in the medium. The highest titer of 32.32 ± 0.37 mg l^−1^ was obtained for the strain ST4978, which had *AtPAL2* and *VvVST1* genes under control of pTEF1 promoter, and the other two genes under control of the pPGK1 promoter. Considering that the pTEF1 promoter is somewhat stronger than pPGK1^22^, the results imply that *AtPAL2* and *VvVST1* may be rate limiting. This would also explain the absence of accumulation of pathway intermediates.

### Enhancement of P450 activity

**Figure 2:**
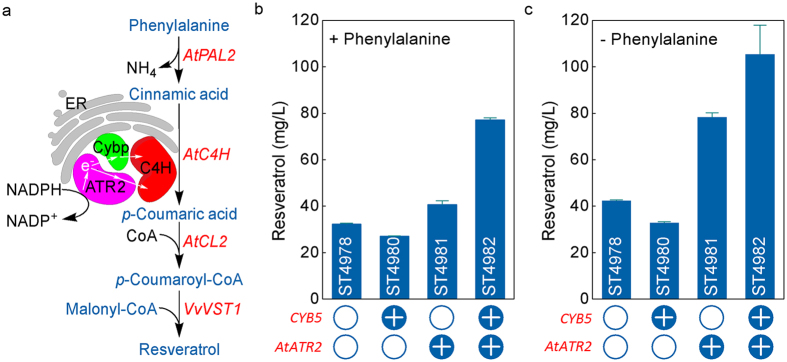
(**a**) Cytochrome P450 reductase (CPR)-mediated electron transfer from NADPH to cinnamic acid hydroxylase (*AtC4H*) and effects of functional expression of CPR (*AtATR2*) and cytochrome B5 (*CYB5*) on resveratrol production on mineral medium with (**b**) and without (**c**) 5 mM phenylalanine in 96-deep-well plates. The displayed average values and standard deviations were calculated from three biological replicates.

Many cytochrome P450 monooxygenases present a challenge, when expressed in a heterologous host, due to their notorious low activity, limited stability, NAD(P)H-dependence, and auxiliary of electron carrier proteins^23^. As C4H used in resveratrol pathway is a membrane-associated plant-derived P450 enzyme, which requires an electron carrier for optimal activity, we hypothesized that C4H activity may be limiting resveratrol production. Plant-derived P450s have previously been reported to poorly accept electrons directly from yeast endogenous electron carriers^24^. Thus, we upgraded the strain ST4978 (basic resveratrol pathway) with overexpression of cytochrome P450 reductase (*AtATR2*) from *A. thaliana* or cytochrome B5 (*CYB5*) from *S. cerevisiae* or both (Fig. 2). Overexpression of *CYB5* did not improve resveratrol production on mineral medium with 5 mM phenylalanine supplementation (Fig. 2b, ST4980), possibly due to inability to directly donate electrons to C4H and due to induction of membrane proliferation^25^. On the other hand, resveratrol titer increased from 32.32 ± 0.37 to 40.75 ± 1.60 mg l^−1^ when *AtATR2* was introduced (Fig. 2b, ST4981), and further increased to 77.19 ± 0.84 mg l^−1^ when *CYB5* was subsequently overexpressed (Fig. 2b, ST4982), which corresponds to 26% and 139% improvement in relation to the reference strain ST4978. Trace amount of *p*-coumaric acid was also detected during the cultivation process (at 24 h) of the strain ST4982, but not in the other strains. Thus, the activity of C4H was clearly enhanced when ATR2 and Cyb5p were overexpressed.

We then tested resveratrol production from glucose by the engineered strains on mineral medium without supplementation of phenylalanine. Interestingly, higher resveratrol production was obtained for all the strains without phenylalanine supplementation (Fig. 2c). This shows that resveratrol can be *de novo* synthesized from glucose via the phenylalanine pathway and that adding phenylalanine to the medium inhibits resveratrol production. The inhibition effect of phenylalanine was more obvious when *AtATR2* was expressed (Fig. 2b,c). ST4981 and ST4982 resulted in 78.30 ± 1.93 mg l^−1^ and 105.31 ± 12.59 mg l^−1^ resveratrol respectively on mineral medium without phenylalanine, which was 92% and 36% higher than resveratrol production with phenylalanine supplementation. We also observed that, with the increase of phenylalanine concentration from 0 to 5 mM, the biomass accumulation increased (Supplementary Fig. 1).

### Increasing copy number of the resveratrol pathway genes

**Figure 3:**
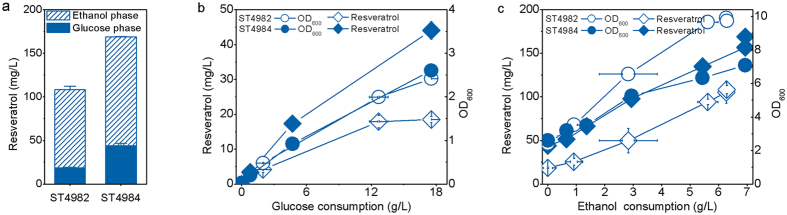
(**a**) Resveratrol production from glucose by strains carrying single and multiple copies of resveratrol pathway. Resveratrol titer in relation to substrate consumption in glucose phase (**b**) and ethanol phase (**c**) by the engineered strains. The strains were cultivated on mineral medium with 20 g l^−1^ glucose in shake flasks. The displayed average values and standard deviations were calculated from three biological replicates.

We have previously shown that for tyrosine-mediated resveratrol biosynthesis the production of resveratrol could be increased 36-fold by integration of multiple copies of the biosynthetic genes^20^. We therefore tested whether the same pull-strategy would work for the phenylalanine-mediated pathway. The basic resveratrol biosynthesis pathway genes (*AtPAL2, AtC4H, At4CL2* and *VvVST1*) were integrated onto Ty-elements in the *AtATR2* and *CYB5* overexpression strain. The weakened *URA3* marker ensures multiple integrations of the expression cassette^26^. 12 isolates were screened to identify the highest producing strain ST4984, which had 2 copies of the resveratrol pathway genes when checked by qPCR. When the strain ST4984 was cultivated on mineral medium with 2% glucose in shake flasks, 169.04 ± 2.42 mg l^−1^ resveratrol was obtained (Fig. 3a). In contrast, only 108.31 ± 4.68 mg l^−1^ resveratrol was obtained with strain ST4982 carrying a single copy of the resveratrol pathway. Resveratrol production was growth associated (Fig. 3b,c). About 17% and 26% of the total resveratrol were produced during the growth on glucose for ST4982 and ST4984 respectively, while the majority of resveratrol was produced during growth on ethanol for both strains (Fig. 3a). One possible explanation for the higher production of resveratrol in the ethanol consumption phase can be increased availability of cytosolic acetyl-CoA, which is an intermediate in ethanol assimilation. This is consistent with the results we have obtained in our previous study^20^. The effect of the multicopy gene integration was however mostly pronounced in the glucose phase, where 2.4-fold more resveratrol was produced in ST4984 than in ST4982. For the ethanol phase the relative increase was smaller, namely 1.4-fold. Interestingly the multicopy strain ST4984 had a lower biomass yield on ethanol than ST4982, while the biomass yields on glucose were similar (Fig. 3b). The final OD_600_ of ST4984 was 30% lower than that of ST4982 (Fig. 3c) probably due to the metabolic burden brought by the high-level expression of the resveratrol pathway enzymes.

### Precursor supply

**Figure 4:**
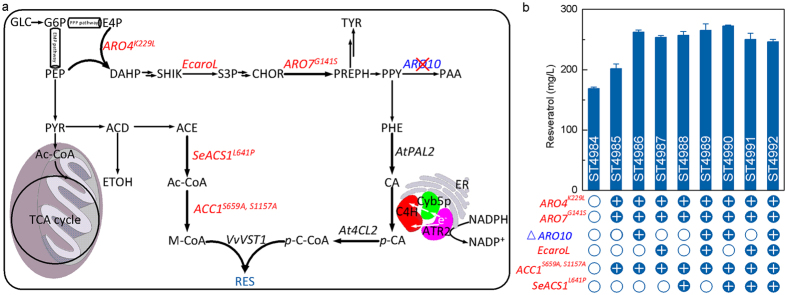
(**a**) Schematic overview of resveratrol biosynthesis in engineered yeast *S. cerevisiae* and the main engineering targets implemented in this study. Single arrows represent single reaction steps, while two arrows represent multiple reaction steps. The modified targets are shown in red, while the gene *ARO10* subjected to deletion is shown in blue. Arrows shown in bold indicate that the genes were overexpressed. GLC, glucose; G6P, glucose 6-phosphate; E4P, erythrose 4-phosphate; DAHP, 3-deoxy-D-arabino-heptulosonic acid 7-phosphate, SHIK, shikimate; S3P, shikimate 3-phosphate; CHOR, chorismate; PREPH, prephenate; TYR, tyrosine; PPY, phenylpyruvate; PAA, phenylacetaldehyde; PHE, phenylalanine; CA, cinnamic acid; *p*-CA, *p*-coumaric acid; *p*-C-CoA, *p*-coumaroyl-CoA; RES, resveratrol; M-CoA, malonyl-CoA; Ac-CoA, acetyl-CoA; ACE, acetate; ACD, acetaldehyde; ETOH, ethanol; PYR, pyruvate; PEP, phosphoenolpyruvate; *ARO4*^*K229L*^, feedback-inhibition resistant version of DAHP synthase; *ARO7*^*G141S*^, feedback-inhibition resistant version of chorismate mutase; *EcaroL, E. coli* shikimate kinase II; *ARO10*, phenylpyruvate decarboxylase, *AtPAL2*, phenylalanine ammonia lyase; *AtC4H*, cinnamate-4-hydroxylase; *At4CL2*, 4-coumarate-CoA ligase; *VvVST1*, resveratrol synthase; *SeACS*^*L641P*^, post-translationally de-regulated variant of acetyl-CoA synthetase; *ACC1*^*S659A, S1157A*^, acetyl-CoA carboxylase devoid of *SNF1*-phosphorylation sites. (**b**) Microbial production of resveratrol from glucose by strains with different genetic modifications. The strains were cultivated on mineral medium with 20 g l^−1^ glucose in shake flasks. The displayed average values and standard deviations were calculated from three biological replicates.

To further improve resveratrol production we applied a push-and-block strategy, where we overexpressed the upstream pathways and eliminated competing pathways for the precursor phenylalanine. To increase precursors supply, the feedback-inhibition resistant versions of DAHP synthase (*ARO*4^*K229L*^) and chorismate mutase (*ARO*7^*G141S*^)^27^ and a de-regulated variant of acetyl-CoA carboxylase (*ACC1*^*S659A, S1157A*^)^28^ were overexpressed in ST4984 to generate ST4985. The strain ST4985 resulted in a 19% improvement of resveratrol production to 201.72 ± 7.91 mg l^−1^ (Fig. 4b), which is supported by our previous study^20^. The strain ST4985 was further modified by the following strategies and combinations thereof in order to increase the push by overproduction of precursors: (i) deletion of phenylpyruvate decarboxylase (*ARO10*), (ii) overexpression of a heterologous shikimate kinase (*aroL*) from *E. coli*, (iii) overexpression of a post-translationally non-regulated version of acetyl-CoA synthase (*SeACS*^*L641P*^) from *Salmonella enterica*^29^. While each of the tested strategies individually resulted in 26–30% increase of resveratrol titer, their effects was not additive upon combination (Fig. 4b). No by-products, such as *p*-coumaric acid or cinnamic acid, were observed at the end of cultivation process in any of the strains. The highest production of resveratrol (272.64 ± 1.34 mg l^−1^) was obtained in strain ST4990, in which *ARO10* was deleted and *SeACS*^*L641P*^ was overexpressed.

### Fed-batch fermentation

**Figure 5:**
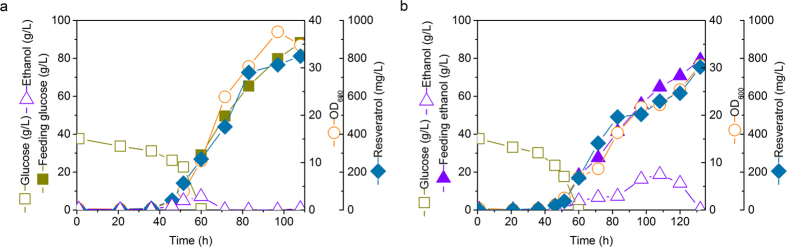
Fed-batch fermentation of the strain ST4990. Aerobic fed-batch fermentations were carried out by feeding glucose (**a**) or ethanol (**b**) respectively with a constant feeding rate of 5 g h^−1^ or 10 g h^−1^.

Fed-batch fermentation of the engineered strain ST4990 was performed in controlled bioreactors on mineral medium with glucose (Fig. 5a) or ethanol (Fig. 5b) feed in the feeding phase. The batch phase was on 40 g l^−1^ glucose, once the glucose was consumed, the constant feeding of glucose or ethanol was initiated. A long lag phase of 40 h was observed followed by a fast growing log phase with the rapid consumption of glucose (Fig. 5). Resveratrol accumulation was growth-associated and reached 268.70 mg l^−1^ with a yield of 11.35 mg g^−1^ glucose when glucose was depleted. A nearly linear increase in OD_600_ over time was observed while glucose or ethanol was being fed. The OD_600_ increase and resveratrol accumulation corresponded well with the substrate consumption (Fig. 5a,b). At the end of the glucose fed-batch fermentation, 812 mg l^−1^ resveratrol and 22 g l^−1^ dry weight (DW) biomass was obtained from feeding 88 g l^−1^ glucose. In the ethanol fed-batch fermentation, where 79 g l^−1^ ethanol was fed, the final concentrations of DW biomass (19 g l^−1^) and resveratrol (755 mg l^−1^) were similar to the reactors with glucose feeding strategy.

### Production of resveratrol derivatives

**Figure 6:**
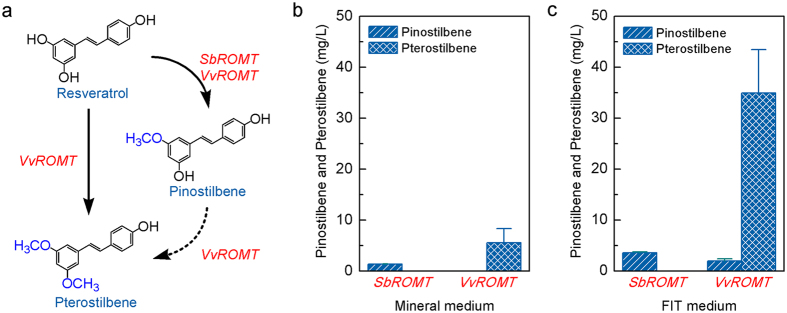
(**a**) Methylation of resveratrol to produce its derivatives, pinostilbene and pterostilbene, by strains ST4993 and ST4994 carrying *SbROMT* and *VvROMT* respectively. The strains were cultivated on mineral medium with 20 g l^−1^ glucose (**b**) or on FIT medium (**c**) in shake flasks. The displayed average values and standard deviations were calculated from duplicates.

The instability of resveratrol, which is sensitive to light and oxygen, limits the bioavailability and bioactivity of the compound^30^. The bioactivity and bioavailability of resveratrol can be enhanced by substitution of hydroxyl groups with methoxy groups^31^,^32^. Two resveratrol O-methyltransferases from *Sorghum bicolor* (*SbROMT*) and *Vitis vinifera* (*VvROMT*) were shown to methylate resveratrol to pinostilbene and pterostilbene, respectively (Fig. 6a)^11^,^13^. We expressed the two enzymes in the resveratrol producing strain ST4990 to generate strains ST4993 and ST4994. Growing the strains ST4993 and ST4994 on mineral medium with 20 g l^−1^ glucose resulted in 1.38 ± 0.06 mg l^−1^ pinostilbene and 5.52 ± 2.84 mg l^−1^ pterostilbene, respectively (Fig. 6b). When grown on feed-in-time (FIT) medium, the strain ST4993 accumulated 5.52 ± 2.84 mg l^−1^ pinostilbene and strain ST4994 accumulated 34.93 ± 8.53 mg l^−1^ pterostilbene (Fig. 6c). Interestingly, 1.96 ± 0.42 mg l^−1^ pinostilbene was also observed in ST4994 culture (Fig. 6c), which indicates that the two methylation groups are introduced sequentially, as also proposed before by Wang *et al*.^13^. Although only small amounts of resveratrol derivatives were detected, it demonstrates for the first time the feasibility of *de novo* biosynthesis of the two resveratrol derivatives from glucose.

## Discussion

We have applied a pull-push-block strategy to improve resveratrol production. The “pull” was improved by optimizing the resveratrol pathway expression and P450 function, the “push” was achieved by increasing the supply of precursors phenylalanine and malonyl-CoA, and finally the “block” was realized by reducing the degradation of the pathway intermediates.

In earlier reports on resveratrol production via phenylalanine, the cultures were supplemented with phenylalanine. For example, Trantas *et al*. have reported a yeast strain that produced 0.29 mg l^−1^ resveratrol when 10 mM phenylalanine was fed to the medium^18^. We found that supplementation of the medium with as little as 0.25 mM phenylalanine decreased resveratrol titer. This phenomena could be explained by the inhibitory effect of phenylalanine on C4H^33^. We previously succeeded in inserting up to 8 copies of the resveratrol pathway (TAL) onto yeast genome^20^. However, here where we produce resveratrol via phenylalanine we managed to integrate only 2 copies of the genes encoding the resveratrol pathway enzymes. The failure to obtain integration of more copies of the genes may result from heavy stress response, such as ER membrane proliferation^34^ or ER morphology variation^35^, when the P450 enzyme is highly expressed. Another plausible explanation might be that the yeast host would incur too much metabolic burden if the copy number of resveratrol pathway integrated onto the genome were very high and this causes a major shift in protein allocation, which has recently been shown to have significant impact on yeast metabolism^36^. The decreased yield of biomass on glucose that was observed in the multicopy strain is consistent with this hypothesis. In addition, we also observed that accumulation of resveratrol was strongly related to growth, which is also supported by recently published study on requirement of ATP for resveratrol production^37^.

Overexpression of feedback-inhibition resistant versions of DAHP synthase (*ARO4*^*K229L*^) and chorismate mutase (*ARO7*^*G141S*^) together with constitutively active acetyl-CoA carboxylase (*ACC1*^*S659A, S1157A*^) gave a 19% improvement in resveratrol titer, which is similar to the increase we observed in the previous study for TAL pathway^20^. We further improved phenylalanine supply by inactivating phenylpyruvate decarboxylase. Two broad-substrate-specificity decarboxylases, Aro10p and Pdc5p, were reported to catalyze decarboxylation of phenylpyruvate to phenylacetaldehyde, i.e., the first degradation step^38^. Although a previous study showed that double deletion of *ARO10* and *PDC5* improved *p*-coumaric acid production^39^, no further improvement of resveratrol production was obtained when *PDC5* was knocked out in addition to *ARO10* (Supplementary Fig. 2). This is consistent with publication by Vuralhan *et al*., who found that Aro10p had higher activity than Pdc5p towards phenylpyruvate^40^. In addition, the supply of malonyl-CoA, another key precursor for resveratrol, would be attenuated when *PDC5* was knocked out as Pdc5p is also responsible for pyruvate decarboxylation. We have previously obtained negative results upon combined overexpression of *ScACS*^*L641P*^ and *ALD6*^20^. On the other hand, overexpression of *ALD6* may lead to reduction of phenylalanine supply through the Ehrlich pathway^38^. Consequently, in this study we chose to overexpress *SeACS*^*L641P*^ alone, which resulted in improvement of resveratrol production. Further strategies for increasing malonyl-CoA supply could be envisioned, such as down-regulation of the competing pathway towards fatty acid biosynthesis^19^.

While the final strain harbored a dozen of genetic modifications, the resveratrol yield (0.007 mol mol^−1^ glucose) was still far lower than the maximum theoretical yield of 0.28 mol mol^−1^ glucose^37^, which shows that there is a lot of potential to further improve the strain. One of the strategies that would be interesting to test could be optimization of the energetics of cytosolic acetyl-CoA generation by overexpression of bacterial pyruvate dehydrogenase complex in the cytosol as recently reported by Kozak *et al*.^41^. Besides, strong correlation between resveratrol biosynthesis and biomass indicates that low biomass is another issue to be solved for improving resveratrol production. Therefore, decoupling growth and production as illustrated recently using a biosensor for malonyl-CoA^42^ could possibly further increase the production of resveratrol.

## Methods and Materials

### Strains and plasmids

All the engineered yeast strains (Table 1) were constructed from CEN.PK102-5B (MATa *ura*3-52 *his*3Δ1 *leu*2-3/112 *MAL*2-8^*c*^ *SUC*2)^43^. Genetic engineering was carried out using either integrative EasyClone vectors with auxotrophic selection markers^44^ or using EasyClone-MarkerFree vectors with CRISPR/Cas9 system^45^. Details on the cloning and strain construction are provided in Supplementary Information. All the oligos used for genetic modifications are listed in Supplementary Table 1. All the biobricks and plasmids used in the study are summarized in Supplementary Tables 2 and 3 respectively.

### Media and cultivations

Seed cultures were prepared by cultivating yeast in SC Drop-out (SD) liquid medium without histidine, leucine and uracil at 30 °C with 250 rpm agitation for 24 h. The inoculation size of 10% (v/v) for 96-deep well plate cultivation or initial OD_600_ of 0.02 for shake flask cultivation was used. The mineral medium (pH 6.0) with 2% glucose or feed-in-time (FIT) medium (m2p-labs, Inc.) and cultivation conditions for resveratrol and its derivatives production was described in ref. 20. Samples were taken at regular intervals or at the end of cultivation. OD was measured at a wavelength of 600 nm using a Genesys 20 spectrophotometer (Thermo Scientific). The dry cell weight was measured by filtrating 3 ml of the cultures through membrane filters and drying at 105 °C to a constant weight. Part of the sample was centrifuged at 12,000 rpm for 2 min and the supernatant was used for analysis of general secreted metabolites. Another part of the sample was mixed with an equal volume of ethanol (99.9%), centrifuged at 12,000 rpm for 2 min and the supernatant was used for resveratrol quantification.

### HPLC and LC-MS measurements

Glucose and ethanol concentrations were quantified by HPLC (Thermo Fisher Scientific, CA) equipped with an Aminex HPX-87H ion-exchange column (Bio-Rad, Hercules, CA) and a UV and RI detector. 5 mM H_2_SO_4_ was used as the mobile phase and the column was kept at 45 °C with a flow rate of 0.6 ml min^−1^. The HPLC detection was carried out with 10 mM ammonium formate (pH 3.0) and acetonitrile as the eluents at a linear gradient flow rate of acetonitrile from 5% to 60% with a Discovery HS F5 150 mm × 2.1 mm column (particle size 3 mm) as described in ref. 20. The analyses of pinostilbene and pterostilbene were performed by LC-MS (Thermo Fisher Scientific, CA). Confirmation of the identity of pinostilbene and pterostilbene was done by comparing the retention time and accurate mass spectrum with the standards purchased from Sigma-Aldrich. The details on LC-MS analysis are provided in Supplementary Information.

### Fed-batch fermentation

Fermentation was carried out in Sartorius bioreactors equipped with an acoustic gas analyzer (model number 1311, Bruël & Kjær). An initial OD_600_ of 0.2 was used for inoculation of seed culture into 0.4 l mineral medium containing 4% glucose. During the fermentation the temperature was maintained at 30 °C, pH at 6.0 with NH_4_·H_2_O, agitation rate at 800 rpm, and air flow at 1 l min^−1^. The detailed setup for batch and fed-batch fermentation is described in Supplementary Information.

## Additional Information

**How to cite this article**: Li, M. *et al*. Engineering yeast for high-level production of stilbenoid antioxidants. *Sci. Rep.* **6**, 36827; doi: 10.1038/srep36827 (2016).

**Publisher’s note:** Springer Nature remains neutral with regard to jurisdictional claims in published maps and institutional affiliations.

## Supplementary Material

Supplementary Information

## Figures and Tables

**Table 1:** List of yeast strains used in the study.

Strains	Genotype	Reference
ST4976	P_*PGK1*_->*AtPAL2*, P_*TEF1*_->*AtC4H*, P_*PGK1*_->*At4CL2*, P_*TEF1*_->*VvVST1*	This study
ST4977	P_*PGK1*_->*AtPAL2*, P_*TEF1*_->*AtC4H*, P_*TEF1*_->*At4CL2*, P_*PGK1*_->*VvVST1*	This study
ST4978	P_*TEF1*_->*AtPAL2*, P_*PGK1*_->*AtC4H*, P_*PGK1*_->*At4CL2*, P_*TEF1*_->*VvVST1*	This study
ST4979	P_*TEF1*_->*AtPAL2*, P_*PGK1*_->*AtC4H*, P_*TEF1*_->*At4CL2*, P_*PGK1*_->*VvVST1*	This study
ST4980	P_*TEF1*_->*AtPAL2*, P_*PGK1*_->*AtC4H*, P_*PGK1*_->*At4CL2*, P_*TEF1*_->*VvVST1*, P_*TEF1*_->*CYB5*	This study
ST4981	P_*TEF1*_->*AtPAL2*, P_*PGK1*_->*AtC4H*, P_*PGK1*_->*At4CL2*, P_*TEF1*_->*VvVST1*, P_*TEF1*_->*AtATR2*	This study
ST4982	P_*TEF1*_->*AtPAL2*, P_*PGK1*_->*AtC4H*, P_*PGK1*_->*At4CL2*, P_*TEF1*_->*VvVST1*, P_*TEF1*_->*AtATR2*, P_*PGK1*_->*CYB5*	This study
ST4984	P_*TEF1*_->*AtATR2*, P_*PGK1*_->*CYB5*, Ty-(P_*TDH3*_->*AtPAL2*, P_*FBA1*_->*AtC4H*, P_*PGK1*_->*At4CL2*, P_*TEF1*_->*VvVST1*)	This study
ST4985	P_*TEF1*_->*AtATR2*, P_*PGK1*_->*CYB5*, Ty-(P_*TDH3*_->*AtPAL2*, P_*FBA1*_->*AtC4H*, P_*PGK1*_->*At4CL2*, P_*TEF1*_->*VvVST1*), P_*TEF1*_->*ACC1*^*S659A,*^ ^*S1157A*^, P_*TEF1*_->*ARO7*^*G141S*^, P_*PGK1*_->*ARO4*^*K229 L*^	This study
ST691	MAT*a ura3-52 his3∆1 leu2-3/112 MAL2-8*^*c*^ *SUC2, ΔARO10, ΔPDC5*	39
ST4986	P_*TEF1*_->*AtATR2*, P_*PGK1*_->*CYB5*, Ty-(P_*TDH3*_->*AtPAL2*, P_*FBA1*_->*AtC4H*, P_*PGK1*_->*At4CL2*, P_*TEF1*_->*VvVST1*), P_*TEF1*_->*ACC1*^*S659A,*^ ^*S1157A*^, P_*TEF1*_->*ARO7*^*G141S*^, P_*PGK1*_->*ARO4*^*K229 L*^, *ΔARO10*	This study
ST4995	P_*TEF1*_->*AtATR2*, P_*PGK1*_->*CYB5*, Ty-(P_*TDH3*_->*AtPAL2*, P_*FBA1*_->*AtC4H*, P_*PGK1*_->*At4CL2*, P_*TEF1*_->*VvVST1*), P_*TEF1*_->*ACC1*^*S659A,*^ ^*S1157A*^, P_*TEF1*_->*ARO7*^*G141S*^, P_*PGK1*_->*ARO4*^*K229 L*^, *ΔARO10, ΔPDC5*	This study
ST4987	P_*TEF1*_->*AtATR2*, P_*PGK1*_->*CYB5*, Ty-(P_*TDH3*_->*AtPAL2*, P_*FBA1*_->*AtC4H*, P_*PGK1*_->*At4CL2*, P_*TEF1*_->*VvVST1*), P_*TEF1*_->*ACC1*^*S659A,*^ ^*S1157A*^, P_*TEF1*_->*ARO7*^*G141S*^, P_*PGK1*_->*ARO4*^*K229 L*^, P_*TEF1*_->*EcaroL*	This study
ST4988	P_*TEF1*_->*AtATR2*, P_*PGK1*_->*CYB5*, Ty-(P_*TDH3*_->*AtPAL2*, P_*FBA1*_->*AtC4H*, P_*PGK1*_->*At4CL2*, P_*TEF1*_->*VvVST1*), P_*TEF1*_->*ACC1*^*S659A,*^ ^*S1157A*^, P_*TEF1*_->*ARO7*^*G141S*^, P_*PGK1*_->*ARO4*^*K229 L*^, P_*TDH3*_->*SeACS*^*L641P*^	This study
ST4989	P_*TEF1*_->*AtATR2*, P_*PGK1*_->*CYB5*, Ty-(P_*TDH3*_->*AtPAL2*, P_*FBA1*_->*AtC4H*, P_*PGK1*_->*At4CL2*, P_*TEF1*_->*VvVST1*), P_*TEF1*_->*ACC1*^*S659A,*^ ^*S1157A*^, P_*TEF1*_->*ARO7*^*G141S*^, P_*PGK1*_->*ARO4*^*K229 L*^, *ΔARO10*, P_*TEF1*_->*EcaroL*	This study
ST4990	P_*TEF1*_->*AtATR2*, P_*PGK1*_->*CYB5*, Ty-(P_*TDH3*_->*AtPAL2*, P_*FBA1*_->*AtC4H*, P_*PGK1*_->*At4CL2*, P_*TEF1*_->*VvVST1*), P_*TEF1*_->*ACC1*^*S659A,*^ ^*S1157A*^, P_*TEF1*_->*ARO7*^*G141S*^, P_*PGK1*_->*ARO4*^*K229 L*^, *ΔARO10*, P_*TDH3*_->*SeACS*^*L641P*^	This study
ST4991	P_*TEF1*_->*AtATR2*, P_*PGK1*_->*CYB5*, Ty-(P_*TDH3*_->*AtPAL2*, P_*FBA1*_->*AtC4H*, P_*PGK1*_->*At4CL2*, P_*TEF1*_->*VvVST1*), P_*TEF1*_->*ACC1*^*S659A,*^ ^*S1157A*^, P_*TEF1*_->*ARO7*^*G141S*^, P_*PGK1*_->*ARO4*^*K229 L*^, P_*TEF1*_->*EcaroL*, P_*TDH3*_->*SeACS*^*L641P*^	This study
ST4992	P_*TEF1*_->*AtATR2*, P_*PGK1*_->*CYB5*, Ty-(P_*TDH3*_->*AtPAL2*, P_*FBA1*_->*AtC4H*, P_*PGK1*_->*At4CL2*, P_*TEF1*_->*VvVST1*), P_*TEF1*_->*ACC1*^*S659A,*^ ^*S1157A*^, P_*TEF1*_->*ARO7*^*G141S*^, P_*PGK1*_->*ARO4*^*K229 L*^, *ΔARO10*, P_*TEF1*_->*EcaroL*, P_*TDH3*_->*SeACS*^*L641P*^	This study
ST4993	P_*TEF1*_->*AtATR2*, P_*PGK1*_->*CYB5*, Ty-(P_*TDH3*_->*AtPAL2*, P_*FBA1*_->*AtC4H*, P_*PGK1*_->*At4CL2*, P_*TEF1*_->*VvVST1*), P_*TEF1*_->*ACC1*^*S659A,*^ ^*S1157A*^, P_*TEF1*_->*ARO7*^*G141S*^, P_*PGK1*_->*ARO4*^*K229 L*^, *ΔARO10*, P_*TDH3*_->*SeACS*^*L641P*^, P_*TDH3*_->*SbROMT*	This study
ST4994	P_*TEF1*_->*AtATR2*, P_*PGK1*_->*CYB5*, Ty-(P_*TDH3*_->*AtPAL2*, P_*FBA1*_->*AtC4H*, P_*PGK1*_->*At4CL2*, P_*TEF1*_->*VvVST1*), P_*TEF1*_->*ACC1*^*S659A,*^ ^*S1157A*^, P_*TEF1*_->*ARO7*^*G141S*^, P_*PGK1*_->*ARO4*^*K229 L*^, *ΔARO10*, P_*TDH3*_->*SeACS*^*L641P*^, P_*TDH3*_->*VvROMT*	This study
