# Supplementary material for: Engineering yeast for high-level production of stilbenoid antioxidants
Source: Sci Rep. 2016 Nov 11;6:36827. doi: 10.1038/srep36827 (PMC5105057; doi:10.1038/srep36827)
Supplement: Supplementary Information [file srep36827-s1.pdf]

Engineering yeast for high-level production of stilbenoid antioxidants

Mingji Li[1], Konstantin Schneider[1], Mette Kristensen[1], Irina Borodina[1], & Jens Nielsen[1,2,3*]

Supplemental Material

Biobricks and plasmids construction

All the plasmids were constructed by following the described USER cloning procedure(1). All the biobricks (**Supplementary Table 2**) were amplified using corresponding templates and primers, which are summarized in Supplementary Table 2 and Table 1, respectively. The PCR reactions were performed in PCR instrument (Bio-Rad) using Phu X7 polymerase(2). The generated biobricks were assembled into either EasyClone vectors(1) or EasyClone-MarkerFree vectors (Jessop-Fabre *et al.* submitted) to construct integrative plasmids for gene overexpression. The plasmid pCfB4286 was generated by assembling BB599 (*hph*MXsyn) into opened plasmid pCfB2312 with primers Open_2_fw and Open_2_rv. The constructed plasmids were sequenced by Eurofins Scientific to confirm the correct cloning.

Strain construction

The resveratrol- and its derivatives-producing strains were engineered by transforming corresponding integrative plasmids into the given parent strains (**Supplementary Table 4**). Yeast transformations were performed following lithium acetate protocol(3). When antibiotic selection was used the transformed strains were incubated 30 ℃ for 2 hours prior to plating on selective medium. For CRISPR/Cas9 mediated gene insertions/deletions, the strains were first transformed with Cas9-expressing plasmid pCfB4286 and selected on hygromycin B plates. The resulting Cas9-expressing strains were transformed with appropriate DNA target fragments together with the corresponding gRNA plasmids. The correct genetic modifications were verified by yeast colony PCR using the primers summarized in Supplementary Table 1.

LC-MS measurements

LC-MS measurements were carried out on a Dionex UltiMate 3000 UHPLC (Thermo Fisher Scientific, San Jose, CA) connected to an Orbitrap Fusion Mass Spectrometer (Thermo Fisher Scientific, San Jose, CA). The UHPLC used a Hypersil GOLD PFP, 15 cm x 2.1 mm, 3 μm column. Temperature was 35 ℃ and flow rate was 1 mL/min with a mobile phase of 100% formic acid (0.1%) for 1 min followed by a linear gradient of 100% formic acid (0.1%)/0% acetonitrile (0.1%) to 5% formic acid (0.1%)/95% acetonitrile (0.1%) over 14 minutes. This gradient was held for 2 minute after which it was changed immediately to 100% formic acid (0.1%) and 0% acetonitrile (0.1%) and held for 4 minutes. The sample was passed on to the MS equipped with a heated electrospray ionization source (HESI) in positive-ion mode with nitrogen as nebulizer gas (45 a.u.). The cone and probe temperature were 342 ℃ and 358 ℃, respectively. Probe gas flow was 13 a.u. and spray voltage was 3500 V. Scan range was 100 to 1000 Da and time between scans was 100 ms.

Fed-batch fermentation of yeast strains in controlled reactors

Four seed pre-cultures were prepared by inoculating fresh colonies from agar plate into 5 ml SC (ura^-, his^-, leu^-) medium and cultivating at 30 ℃ for 24 hours in an orbital shaker (250 rpm). The pre-cultures were then inoculated to an initial OD_{600} of 0.02 into 50 ml minimal medium (pH 6.0), as described in(3) in 250 ml flasks. The start OD_{600} of 0.2 in bioreactors was prepared by inoculating seed culture into 400 ml fermentation medium in four bioreactors. The composition of the fermentation medium was as following: 15.0 g l^{-1} $(NH_4)_2SO_4$, 6.0 g l^{-1} KH_2PO_4, 1.0 g l^{-1} MgSO4 $\cdot 7H_2O$, 2 ml l^{-1} trace metals solution, 2 ml l^{-1} vitamins solution, 0.5 ml l^{-1} antifoam A (Sigma-Aldrich), and 40 g l^{-1} glucose, where the composition of trace metal solution and vitamin solution was as described in(1). The medium without vitamins, trace metals and glucose was autoclaved at 121 ℃ for 20 min. Glucose was autoclaved separately using the same conditions.

Vitamins solution and trace metals solution were added to the sterilized medium by filtration. The fermentation was performed at 30 ℃ with the agitation rate of 800 rpm and the air flow of 1 l per min. The pH was automatically maintained at 6.0 with $NH_4 \cdot H_2O$. Once the glucose was depleted, which was judged by the sharp decline of exhaust CO_2, the feed was initialed at a constant feeding rate of 5 g h^{-1} or 10 g h^{-1} for ethanol and glucose feeds respectively. The feed medium contained 45 g l^{-1} $(NH_4)_2SO_4$, 18 g l^{-1} KH_2PO_4, 3 g l^{-1} $MgSO_4 \cdot 7H_2O$, 12 ml L^{-1} trace metals solution, 6 ml L^{-1} vitamins solution, 0.6 ml L^{-1} antifoam A, and 160 g l^{-1} of glucose or ethanol. Glucose, vitamins, and trace metals were added to the feed solution in the same way as to the batch fermentation medium. Samples were taken at regular intervals and used for measurements of biomass and metabolites concentration.

Supplementary Figure 1. Final concentrations of resveratrol and OD_{600} of ST4982, cultivated on minimal medium with 2% glucose and 0 to 5 mM phenylalanine. The strains were grown in 96-deep well plates for 72 hours. The displayed average values and standard deviations were calculated from three biological replicates.

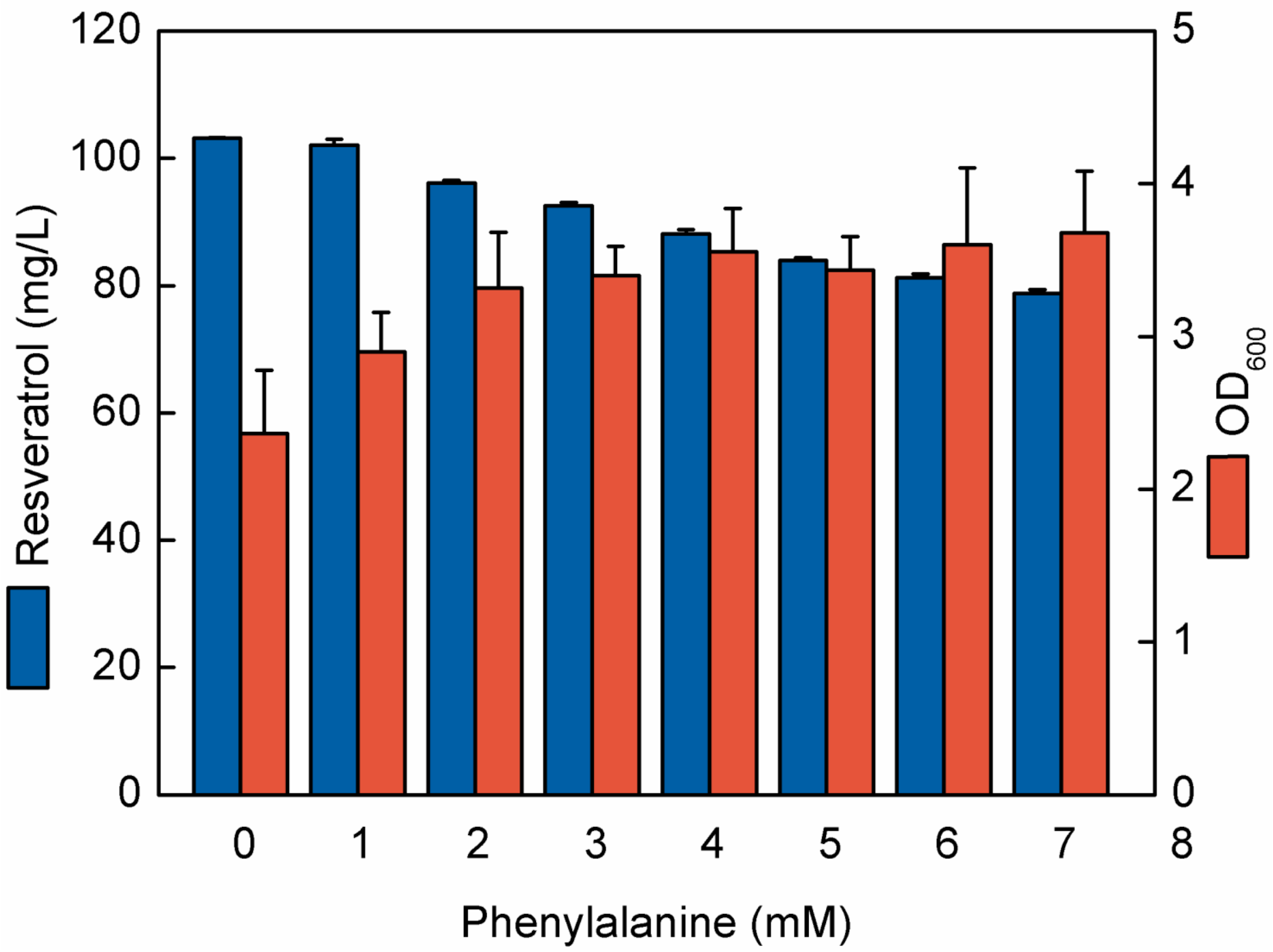

Supplementary Figure 2. Resveratrol production by the strains ST4986 and ST4995 on minimal medium with 2% glucose in shake flasks. The displayed average values and standard deviations were calculated from three biological replicates.

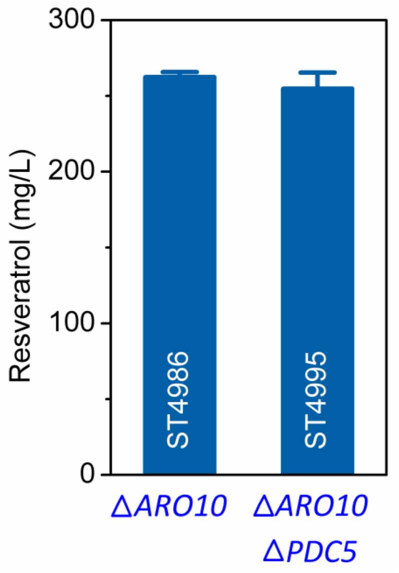

Supplementary Table 1. List of primers used in the study.

Name	Sequence (5’ to 3’)
$P_{<-TEF1}$_fw (ID005)	ACCTGCACU TTGTAATTAAAACTTAG
$P_{<-TEF1}$_rv (ID006)	CACGCGAU GCACACACCATAGCTTC
$P_{PGK1->}$_rv (ID008)	ATGACAGAU TTGTTTTATATTTGTTG
$P_{<-PGK1}$_fw (ID1562)	ACCTGCACU TTGTTTTATATTTGTTG
$P_{TEF1->}$_fw (ID1564)	CGTGCGAU GCACACACCATAGCTTC
$P_{TEF1->}$_rv (ID1565)	ATGACAGAU TTGTAATTAAAACTTAG
<-*AtPAL2*_fw (ID1558)	AGTGCAGGU AAAACAATGGATCAAATC
<-*AtPAL2*_rv (ID1559)	CGTGCGAU TCAGCAGATAGGAATAGG
AtC4H->_fw (ID1546)	ATCTGTCAU AAAACAATGGACTTGTTGTTGTTG
AtC4H->_rv (ID1547)	CACGCGAU TCAACAGTTTCTTGGCTT
<-*CYB5*_fw (ID1495)	AGTGCAGGU AAAACA ATGCCTAAAGTTTACAGTTACC
<-*CYB5*_rv (ID1496)	CGTGCGAU TCA TTCGTTCAACAAATAATAAGC
AtATR2->_fw (ID1554)	ATCTGTCAU AAAACAATGTCCTCCTCTTCTTCATCATCCACC
AtATR2->_rv (ID1555)	CACGCGAU TCACCAGACATCTCTCAA
<-*At4CL2*_fw (ID1550)	AGTGCAGGU AAAACAATGACTACCCAAGATGTTA
<-*At4CL2*_rv (ID1551)	CGTGCGAU TCAGTTCATCAAACCGTT
VvVST1->_fw (ID1552)	ATCTGTCAU AAAACAATGGCTTCCGTTGAAGAA
VvVST1->_rv (ID1553)	CACGCGAU TCAATTGGTAACGGTTGG
Open_1_fw (ID1858)	AGCTGAAGCU TCGTACGCTG
Open_1_rv (ID2167)	ACGCGATCU TCGAGCGTCC
<-*ACC1*_fw (ID53)	CGTGCGAU TCATTTCAAAGTCTTCAACAATTT
<-*ACC1*_rv (ID54)	AGTGCAGGU AAAACAATGAGCGAAGAAAGCTTA
USER_fw (ID400)	ATTGGGU GCATAGGCCACTAGTGGATCTG
USER_rv (ID399)	ATCGCGU CAGCTGAAGCTTCGTACGC
Open_2_fw (ID7078)	ACGCGAU CTCGTGATACGCCTATTTT
Open_2_rv (ID7079)	ACCCAAU ATCAGTTATTACCCTATGCG
TJOS-62 (P1F) (ID 10525)	CGTGCGAU AGGGAACAAAAGCTGGAGCT
TJOS-65 (P1R) (ID 10529)	CACGCGAU TAACTAATTACATGACTCGA
TJOS-64 (P3F) (ID 10527)	ATCTGTCAU AGGGAACAAAAGCTGGAGCT
TJOS-67(P3F) (ID 10531)	ATGACAGAU TAACTAATTACATGACTCGA
*PDC5*_KO_fw (ID1368)	CGTAAACCTGCATTAAG
*PDC5*_KO_rv (ID1371)	CTAAGATCATAGCTAAAGG
*ARO10*_KO_fw (ID1372)	GGATAGCCGTCATTTAC
*ARO10*_KO_rv (ID1375)	CGATAGGAATGACAGAA
EcaroL->_fw (ID6785)	ATCTGTCAU AAAACAATGACACAACCTCTTTTTCTGA
EcaroL->_rv (ID6786)	CACGCGAU TCAACAATTGATCGTCTGTGC
<-*SeACS*_fw (ID644)	AGTGCAGGU AAAACAATGTCACAAACACAC
<-*SeACS*_rv (ID645)	CGTGCGAU TCATGATGGCATAGCAATAG
<-T_{NAT5}_fw (ID2164)	ATCGCACGAU TTCTTAACAGATGGCTG
<-T_{NAT5}_rv (ID2168)	AGATCGCGU TCGGGACCATAAAAATTC
$P_{<-TDH3}$_fw (ID2141)	AGCTTCAGCU ATAAAAAACACGCTTTTTCAG

P$_{\textit{<-TDH3}}$_rv (ID1853)	ACCTGCACU TTTGTTTGTTTATGTGTGTTTATTC
P$_{\textit{FBA1}}$->_fw (ID8489),	GCGTGTTU TTTATATAACAATACTGACAGTACT
P$_{\textit{FBA1}}$->_rv (ID8490)	ATGACAGAU TTTGAATATGTATTACTTGG
*SbROMT*_fw (ID13616)	ATCTGTCAU AAAACAATGGTCTTGATCTCCGAAGATTCC<br>AGAGAATTATTG
*SbROMT*_rv (ID13617)	CACGCGAU TCATGGGTATAATTCGATGAT
*VvROMT*_fw (ID13618)	ATCTGTCAU AAAACAATGGATTTGGCCAAC
*VvROMT*_rv (ID13619)	CACGCGAU TCATGGGTAAACTTCGATCAA
ColPCR_DW_fw (ID2220)	CCTGCAGGACTAGTGCTGAG
X-3_DW_rv (ID904)	CCGTGCAATACCAAAATCG
X-4_DW_rv (ID906)	GACGGTACGTTGACCAGAG
XI-1_DW _rv (ID908)	GAAGACCCATGGTTCCAAGGA
XI-2_DW_rv (ID910)	GAGACAAGATGGGGCAAGAC
XI-5_DW_rv (ID2157)	CCCAAAAGCAATCCAGGAAAAACC
XII-1_DW_rv (ID892)	GGACGACAACTACGGAGGAT
*PDC5*_chk_fw (ID1384)	AAAGCCTCCATATCCAAAG
*PDC5*_chk_rv (ID1385)	AGGTATGGTTAAAGATCACAC
*ARO10*_chk_fw (ID1386)	ACCGAAATTTAAAAAAGCAG
*ARO10*_chk_rv (ID1387)	GTTTTCGGATAAAACTTCTTC

Note: Underlined sequences represent overhangs used for USER cloning.

Supplementary Table 2. List of biobricks used in the study.

Biobrick	Description	Template	Forward primer	Reverse Primer
BB008	Promoter, <-P$_{TEF1}$	pCfB826	P$_{<-TEF1}$_fw	P$_{<-TEF1}$_rv
BB301	Promoter, P$_{TEF1->}$	pCfB826	P$_{TEF1->}$_fw	P$_{TEF1->}$_rv
BB010	Bidirectional promoter, <-P$_{TEF1}$-P$_{PGK1}$->	pCfB826	P$_{<-TEF1}$_fw	P$_{PGK1->}$_rv
BB302	Bidirectional promoter, <-P$_{PGK1}$-P$_{TEF1}$->	pCfB826	P$_{<-PGK1}$_fw	P$_{\mathrm{TEF1->}}$_rv
BB291	*PAL2* from *A. thaliana*	pCfB756	<-*AtPAL2*_fw	<-*AtPAL2*_rv
BB292	*C4H* from *A. thaliana*	pCfB754	*AtC4H*->_fw	*AtC4H*->_rv
BB290	*CYB5* from *S. cerevisiae*	gDNA of CEN.PK102-5B	<-*CYB5*_fw	<-*CYB5*_rv
BB296	*ATR2* from *A. thaliana*	pCfB755	*AtATR2*->_fw	*AtATR2*->_rv
BB294	*4CL2* from *A. thaliana*	pCfB758	<-*At4CL2*_fw	<-*At4CL2*_rv
BB295	*VST1* from *V. vinifera*	pCfB759	*VvVST1*->_fw	*VvVST1*->_rv
BB580	*KanMXpm*	pCfB2055	USER_fw	USER_rv
BB530	Promoter, <-P$_{TDH3}$	gDNA of CEN.PK102-5B	P$_{<-TDH3}$_fw	P$_{<-TDH3}$_rv
BB705	Promoter, P$_{FBA1->}$	gDNA of CEN.PK102-5B	P$_{FBA1->}$_fw	P$_{FBA1->}$_rv
BB713	Promoter, <-P$_{TDH3}$-P$_{FBA1}$->	BB530 + BB705	P$_{<-TDH3}$_fw	P$_{FBA1->}$_rv
BB429	Terminator, T$_{<-NAT5}$	gDNA of CEN.PK102-5B	T$_{<-NAT5}$_fw	T$_{<-NAT5}$_rv
BB1655	Linearized pCfB1178	pCfB1178	Open_1_fw	Open_1_rv
BB599	*hph*MXsyn	pCfB2513	USER_fw	USER_rv
BB364	*ARO4*^{K229L} from *S. cerevisiae*	pCfB826	*ScARO4*->_fw	*ScARO4*->_rv
BB361	*ARO7*^{G141S} from *S. cerevisiae*	pCfB826	<-*ScARO7*_fw	<-*ScARO7*_rv
BB012	*ACC1*$^{S659A, S1157A}$ from *S. cerevisiae*	pCfB1175	<-*ACC1*_fw	<-*ACC1*_rv
BB1268	gBlock of *PDC5*		TJOS-62 (P1F)	TJOS-67 (P3R)
BB1269	gBlock of *ARO10*		TJOS-64 (P3F)	TJOS-65 (P1R)
BB1623	Knockout fragment of *ARO10*	gDNA of ST691	*ARO10*_KO_fw	*ARO10*_KO_rv
BB1644	Knockout fragment of *PDC5*	gDNA of ST691	*PDC5*_KO_fw	*PDC5*_KO_rv
BB501	*aroL* from *E. coli*	pCfB2747	*EcaroL*->_fw	*EcaroL*->_rv
BB119	*ACS*^{L641P} from *S. enterica*	pCfB324	<-*SeACS*_fw	<-*SeACS*_rv
BB1309	*ROMT* from *S. bicolor*	pCfB4659	*SbROMT*->_fw	*SbROMT*->_rv
BB1310	*ROMT* from *V. vinifera*	pCfB4660	*VvROMT*->_fw	*VvROMT*->_rv

Supplementary Table 3. List of plasmids used in the study.

Name	Parent plasmid	Biobricks	Properties	Reference
pCfB756			*AtPAL2*[a], *KanMX*	This study
pCfB754			*AtC4H*[a], *KanMX*	This study
pCfB755			*AtATR2*[a], *KanMX*	This study
pCfB758			*At4CL2*[a], *KanMX*	(4)
pCfB759			*VvVST1*[a], *KanMX*	(4)
pCfB4659			*SbROMT*[a], *KanMX*	This study
pCfB4660			*VvROMT*[a], *KanMX*	This study
pCfB388			Integrative plasmid, XI-1-LoxP, *KlLEU2*	(1)
pCfB389			Integrative plasmid, XI-2-LoxP, *KlURA3*	(1)
pCfB391			Integrative plasmid, XI-5-LoxP, *SpHIS5*	(1)
pCfB844	pCfB391	BB291, BB302, BB292	Integrative plasmid, XI-5, LoxP, P_{PGK1}-*AtPAL2*, P_{TEF1}-*AtC4H*, *SpHIS5*	This study
pCfB855	pCfB388	BB294, BB302, BB295	Integrative plasmid, XI-1, LoxP, P_{PGK1}-*At4CL2*, P_{TEF1}-*VvVST1*, *KlLEU2*	This study
pCfB846	pCfB389	BB290, BB008,	Integrative plasmid, XI-2, LoxP, P_{TEF1}-*CYB5*, *KlURA3*	This study
pCfB847	pCfB389	IBB301, BB296	Integrative plasmid, XI-2, LoxP, P_{TEF1}-*AtATR2*, *KlURA3*	This study
pCfB848	pCfB389	BB290, BB302, BB296	Integrative plasmid, XI-2, LoxP, P_{PGK1}-*CYB5*, P_{TEF1}-*AtATR2*, *KlURA3*	This study
pCfB1018	pCfB391	BB291, BB010, BB292	Integrative plasmid, XI-5, LoxP, P_{TEF1}-*AtPAL2*, P_{PGK1}-*AtC4H*, *SpHIS5*	This study
pCfB1021	pCfB388	BB294, BB010, BB295	Integrative plasmid, XI-1, LoxP, P_{TEF1}-*At4CL2*, P_{PGK1}-*VvVST1*, *KlLEU2*	This study
pCfB2224			Integrative plasmid, XI-2, LoxP, *KanMX*	(5)
pCfB2767	pCfB2224	BB290, BB302, BB296	Integrative plasmid, XI-2, LoxP, P_{PGK1}-*CYB5*, P_{TEF1}-*AtATR2*, *KanMX*	This study
pCfB826			Integrative plasmid, X-4,LoxP, P_{TEF1}-*ScARO7*^{G141S}, P_{PGK1}-*ScARO4*^{K229L}, *SpHIS5*	(6)
pCfB257			Integrative plasmid, X-3, LoxP, *KlLEU2*	(1)
pCfB2582	pCfB257	BB012, BB008	Integrative plasmid, X-3, LoxP, P_{TEF1}-*ScACC1*$^{S659A, S1157A}$, *KlLEU2*	This study
pCfB322			Multiple integrative plasmid, Ty4, *KlURA3*	(7)
pCfB1178	pCfB322	BB294, BB302,	Multiple integrative plasmid, Ty4, P_{PGK1}-*At4CL2*, P_{TEF1}-*VvVST1*, *KlURA3*	This study

		BB295		
pCfB2584	pCfB1178	BB429, BB291, BB713, BB292	Multiple integrative plasmid, Ty4, P$_{TDH3}$-*AtPAL2*, P $_{FBA1}$-*AtC4H*, P$_{PGK1}$-*At4CL2*, P$_{TEF1}$-*VvVST1*, *KlURA3*	This study
pCfB2312			Centromeric plasmid, P$_{TEF1}$-*CAS9*, *KanMX*.	(5)
pCfB4286	pCfB2312	BB599	Centromeric plasmid, P$_{TEF1}$-*CAS9*, *hph*MXsyn	This study
pTAJAK-71			2 μ plasmid, *NatMX*	(8)
pCfB4156	pCfB2926	BB1268, BB1269	2 μ plasmid, P$_{SNR52}$-gBlock_*PDC5*-gBlock_*ARO10*-T$_{SUP}$, *NatMX*	This study
pCfB3041	pCfB2926	BB1324	2 μ plasmid, P$_{SNR52}$-gBlock_X-3-T$_{SUP}$, *NatMX*	(Jessop-Fabre *et al*., submitted)
pCfB3034			Integrative plasmid, X-3, MarkerFree	(Jessop-Fabre *et al*., submitted)
pCfB2747	pCfB3034		Integrative plasmid, X-3, P$_{PGK1}$-*EcaroL*, *KlLEU2*	(6)
pCfB4289	pCfB3034	BB301, BB501	Integrative plasmid, X-3, P$_{TEF1}$-*EcaroL*, MarkerFree	This study
pCfB3047	pCfB2926		2 μ plasmid, P$_{SNR52}$-gBlock_XII-1-T$_{SUP}$, *NatMX*	(Jessop-Fabre *et al*., submitted)
pCfB3038			Integrative plasmid, XII-1, MarkerFree	(Jessop-Fabre *et al*., submitted)
pCfB4655	pCfB3048	BB704, BB119	Integrative plasmid, XII-1, P$_{TDH3}$-*SeACS*^{L641P}, MarkerFree	This study
			Integrative plasmid, XII-2, MarkerFree	(Jessop-Fabre *et al*., submitted)
pCfB4290		BB704, BB1309	Integrative plasmid, XII-2, P$_{TDH3}$-*SbROMT*, MarkerFree	This study
pCfB4292		BB704, BB1310	Integrative plasmid, XII-2, P$_{TDH3}$-*VvROMT*, MarkerFree	This study

[a] The genes were codon-optimized for *S.cerevisiae* and synthesized by GeneArt (Life Technologies).

The DNA sequences are provided at the end of the Supplementary materials and methods.

Supplementary Table 4. The strains engineered in this study.

Strains	Parent strain	Transformed integrative plasmids
ST4976	CEN.PK102-5B	pCfB844, pCfB855, pCfB389
ST4977	CEN.PK102-5B	pCfB844, pCfB1021, pCfB389
ST4978	CEN.PK102-5B	pCfB1018, pCfB855, pCfB389
ST4979	CEN.PK102-5B	pCfB1018, pCfB1021, pCfB389
ST4980	CEN.PK102-5B	pCfB1018, pCfB855, pCfB846
ST4981	CEN.PK102-5B	pCfB1018, pCfB855, pCfB847
ST4982	CEN.PK102-5B	pCfB1018, pCfB855, pCfB848
ST4984	CEN.PK102-5B	pCfB2767, pCfB2584, pCfB257, pCfB258
ST4985	CEN.PK102-5B	pCfB2767, pCfB2584, pCfB826, pCfB2582
ST4986	ST4985	*ΔARO10*
ST4987	ST4985	pCfB4289
ST4988	ST4985	pCfB4655
ST4989	ST4986	pCfB4289
ST4990	ST4986	pCfB4655
ST4991	ST4985	pCfB4289, pCfB4655
ST4992	ST4986	pCfB4289, pCfB4655
ST4993	ST4990	pCfB4290
ST4994	ST4990	pCfB4292
ST4995	ST4985	*ΔARO10*, *ΔPDC5*

DNA sequence of *AtPAL2* codon-optimized for *S. cerevisiae* by GeneArt (Life Technologies)

ATGGATCAAATCGAAGCTATGTTGTGTGGTGGTGGTGAAAAAACAAAAGTTGCTGTTA
CTACTAAGACCTTGGCCGATCCATTGAATTGGGGTTTGGCTGCTGATCAAATGAAGGGT
TCTCATTTGGATGAAGTCAAGAAGATGGTCGAAGAATACAGAAGACCAGTTGTTAATT
TGGGTGGTGAAACTTTGACTATTGGTCAAGTTGCTGCTATTTCTACTGTTGGTGGTTCTG
TTAAGGTTGAATTGGCTGAAACTTCTAGAGCTGGTGTTAAGGCTTCTTCTGATTGGGTT
ATGGAATCTATGAACAAGGGTACTGATTCTTACGGTGTTACTACAGGTTTTGGTGCTAC
TTCTCATAGAAGAACTAAGAATGGTACTGCCTTGCAAACCGAATTGATCAGATTTTTGA
ACGCCGGTATTTTCGGTAACACCAAAGAAACTTGTCATACCTTGCCACAATCTGCTACT
AGAGCTGCTATGTTGGTTAGAGTTAACACTTTGTTGCAAGGTTACTCCGGTATCAGATT
CGAAATTTTGGAAGCTATCACCTCCTTGTTGAACCATAACATTTCTCCATCTTTGCCATT
GAGAGGTACTATTACTGCTTCTGGTGATTTGGTTCCATTGTCTTATATTGCTGGTTTGTT
GACTGGTAGACCAAACTCTAAAGCTACTGGTCCAGATGGTGAATCATTGACTGCTAAA
GAAGCTTTTGAAAAGGCTGGTATCTCTACTGGTTTTTTCGACTTGCAACCTAAAGAAGG
TTTGGCTTTGGTTAATGGTACAGCTGTTGGTTCTGGTATGGCTTCTATGGTTTTGTTTGA
AGCTAACGTTCAAGCTGTTTTGGCCGAAGTTTTGTCTGCTATTTTTGCTGAAGTTATGTC
CGGTAAGCCAGAATTCACTGATCATTTGACCCATAGATTGAAACATCACCCAGGTCAA
ATTGAAGCTGCTGCAATTATGGAACATATCTTGGATGGTTCCTCTTACATGAAGTTGGC
TCAAAAAGTTCACGAAATGGACCCATTGCAAAAGCCAAAACAAGATAGATACGCTTTG
AGAACTTCTCCACAATGGTTGGGTCCACAAATAGAAGTTATTAGACAAGCCACCAAGT
CCATCGAAAGAGAAATCAATTCTGTTAACGACAACCCATTGATCGACGTCAGTAGAAA
CAAAGCTATTCATGGTGGTAACTTCCAAGGTACTCCAATTGGTGTTTCTATGGACAACA
CTAGATTGGCTATTGCTGCCATTGGTAAATTGATGTTCGCTCAATTCTCCGAATTGGTC
AACGATTTTTACAACAACGGTTTGCCTTCTAACTTGACCGCTTCTTCTAATCCATCATTG
GATTACGGTTTTAAGGGTGCTGAAATTGCTATGGCTTCATACTGTTCTGAATTGCAATA
CTTGGCTAACCCAGTTACCTCTCATGTTCAATCTGCTGAACAACACAATCAAGACGTTA
ACTCCTTGGGTTTGATCTCTTCTAGAAAGACTTCTGAAGCCGTTGACATCTTGAAGTTG
ATGTCTACTACATTCTTGGTCGGTATTTGCCAAGCTGTTGATTTGAGACATTTGGAAGA
AAACTTGAGACAAACCGTCAAGAACACCGTTTCACAAGTTGCTAAGAAAGTTTTGACC
ACCGGTATTAACGGTGAATTGCATCCATCTAGATTCTGCGAAAAGGATTTGTTGAAGGT
CGTTGATAGAGAACAAGTTTTCACCTACGTTGATGATCCATGTTCTGCTACTTATCCAT
TGATGCAAAGATTGAGACAAGTCATCGTTGATCATGCTTTGTCTAATGGTGAAACCGA
AAAGAACGCTGTTACCTCCATTTTCCAAAAGATTGGTGCTTTCGAAGAAGAATTGAAG
GCCGTTTTGCCAAAAGAAGTTGAAGCAGCTAGAGCAGCTTACGGTAACGGTACTGCTC
CAATTCCAAATAGAATCAAAGAATGCAGATCCTACCCATTATACAGATTCGTTAGAGA
AGAATTAGGTACTAAGTTGTTGACCGGTGAAAAGGTTGTTTCTCCAGGTGAAGAATTC
GATAAGGTTTTCACTGCTATGTGCGAAGGTAAATTGATCGATCCATTGATGGACTGCTT
GAAAGAATGGAATGGTGCTCCTATTCCTATCTGCTGA

DNA sequence of *AtC4H* codon-optimized for *S. cerevisiae* by GeneArt (Life Technologies)

ATGGACTTGTTGTTGTTGGAAAAGTCCTTGATTGCTGTTTTCGTTGCTGTTATTTTGGCC
ACCGTTATCTCTAAATTGAGAGGTAAGAAATTGAAGTTGCCACCAGGTCCAATTCCAA
TCCCAATTTTTGGTAATTGGTTGCAAGTTGGTGATGACTTGAACCACAGAAACTTGGTT
GATTACGCTAAAAAGTTCGGTGATTTGTTCTTGTTGAGAATGGGTCAAAGAAATTTGGT
CGTTGTTTCCTCACCAGACTTGACCAAAGAAGTTTTGTTGACTCAAGGTGTCGAATTCG
GTTCCAGAACTAGAAATGTTGTTTTCGATATCTTCACCGGTAAGGGTCAAGATATGGTT
TTTACTGTTTACGGTGAACATTGGAGAAAGATGAGAAGAATTATGACCGTTCCATTCTT
CACCAACAAGGTTGTCCAACAAAACAGAGAAGGTTGGGAATTTGAAGCTGCTTCTGTT
GTTGAAGATGTCAAGAAGAATCCAGATTCTGCTACTAAGGGTATCGTTTTGAGAAAAA
GATTGCAATTGATGATGTACAACAACATGTTCAGAATCATGTTCGACAGAAGATTTGA
ATCCGAAGATGACCCTTTGTTTTTGAGATTGAAGGCTTTGAACGGTGAAAGATCTAGAT
TGGCTCAATCCTTCGAATACAACTACGGTGATTTCATCCCAATCTTAAGACCATTCTTG
AGAGGTTACTTGAAGATCTGCCAAGATGTTAAGGATAGAAGAATCGCCTTGTTCAAAA
AGTACTTCGTTGACGAAAGAAAGCAAATCGCTTCTTCTAAACCTACTGGTTCTGAAGGT
TTGAAGTGCGCCATTGATCATATTTTGGAAGCTGAACAAAAGGGTGAAATCAACGAAG
ATAACGTCTTGTACATCGTCGAAAACATTAACGTTGCTGCTATTGAAACTACCTTGTGG
TCTATTGAATGGGGTATTGCTGAATTGGTTAATCACCCAGAAATCCAATCCAAGTTGAG
AAACGAATTGGATACTGTTTTGGGTCCAGGTGTTCAAGTTACTGAACCTGACTTGCATA
AGTTGCCATACTTGCAAGCTGTTGTAAAAGAAACCTTGAGATTAAGAATGGCCATCCC
TTTGTTGGTTCCACATATGAACTTGCATGATGCTAAATTGGCCGGTTATGATATTCCAG
CCGAATCCAAGATTTTGGTTAATGCTTGGTGGTTGGCTAACAATCCAAATTCTTGGAAA
AAGCCAGAAGAATTCAGACCAGAAAGATTTTTCGAAGAAGAAAGTCACGTTGAAGCC
AACGGTAATGATTTTAGATACGTTCCATTTGGTGTTGGTAGAAGATCTTGTCCAGGTAT
TATCTTGGCTTTGCCAATTTTGGGTATTACCATCGGTAGAATGGTCCAAAACTTCGAAT
TATTGCCACCACCTGGTCAATCTAAGGTTGATACTTCTGAAAAGGGTGGTCAATTCTCC
TTGCATATTTTGAACCACTCCATCATCGTTATGAAGCCAAGAAACTGTTGA

DNA sequence of *AtATR2* codon-optimized for *S. cerevisiae* by GeneArt (Life Technologies)

ATGTCCTCCTCTTCTTCATCATCCACCTCTATGATTGATTTGATGGCCGCTATTATCAAG
GGTGAACCAGTTATAGTTTCTGATCCAGCTAATGCTTCTGCCTATGAATCTGTTGCTGC
TGAATTATCCTCCATGTTGATCGAAAACAGACAATTCGCTATGATCGTCACTACCTCTA
TTGCTGTTTTGATTGGTTGCATCGTTATGTTGGTTTGGAGAAGATCTGGTTCTGGTAACT
CTAAAAGAGTCGAACCATTGAAGCCATTGGTTATCAAACCTAGAGAAGAAGAAATTGA
CGACGGTAGAAAGAAGGTTACCATTTTCTTTGGTACTCAAACCGGTACTGCTGAAGGTT
TTGCTAAAGCTTTGGGTGAAGAAGCTAAAGCCAGATACGAAAAGACTAGATTCAAGAT
CGTTGACTTGGATGATTACGCTGCAGATGATGATGAATACGAAGAAAAGTTGAAGAAA
GAAGATGTCGCCTTTTTCTTCTTGGCTACTTATGGTGATGGTGAACCTACTGATAATGC
TGCTAGATTTTACAAGTGGTTCACCGAAGGTAATGATAGAGGTGAATGGTTGAAAAAC
TTGAAGTACGGTGTTTTCGGTTTGGGTAATAGACAATACGAACACTTCAACAAGGTTGC
CAAGGTTGTTGATGATATCTTGGTTGAACAAGGTGCCCAAAGATTGGTTCAAGTTGGTT
TAGGTGATGATGACCAATGCATCGAAGATGATTTTACTGCTTGGAGAGAAGCTTTGTG
GCCAGAATTGGATACAATCTTGAGAGAAGAAGGTGATACTGCTGTTGCTACTCCATAT
ACTGCTGCTGTTTTAGAATACAGAGTTTCCATCCACGATTCCGAAGATGCTAAGTTCAA
CGATATTAACATGGCTAACGGTAACGGTTACACCGTTTTTGATGCTCAACATCCATACA
AGGCTAACGTTGCTGTTAAGAGAGAATTGCATACTCCAGAATCTGACAGATCCTGCAT
TCATTTGGAATTCGATATTGCTGGTTCCGGTTTGACTTACGAAACTGGTGATCATGTTG
GTGTTTTGTGCGATAACTTGTCTGAAACTGTTGATGAAGCCTTGAGATTATTGGATATG
TCTCCAGATACCTACTTCTCCTTGCATGCCGAAAAAGAAGATGGTACTCCAATCTCTTC
ATCTTTGCCACCACCATTTCCACCATGTAATTTGAGAACTGCTTTGACCAGATACGCTT
GCTTGTTGTCATCTCCAAAAAAGTCTGCTTTGGTTGCTTTGGCTGCTCATGCTTCAGATC
CAACTGAAGCTGAAAGATTGAAACATTTGGCTTCTCCAGCTGGTAAGGATGAATATTC
TAAATGGGTTGTTGAATCCCAAAGATCCTTGTTGGAAGTTATGGCTGAATTTCCATCTG
CTAAACCACCATTGGGTGTTTTTTTTGCTGGTGTTGCTCCAAGATTGCAACCTAGATTCT
ACTCTATTTCCTCCTCCCCAAAAATTGCCGAAACCAGAATTCATGTTACTTGCGCTTTG
GTCTACGAAAAAATGCCAACTGGTAGAATCCATAAGGGTGTTTGTTCTACCTGGATGA
AGAATGCTGTTCCTTACGAAAAGTCCGAAAACTGTTCTTCTGCTCCAATCTTCGTTAGA
CAATCCAATTTCAAGTTGCCATCCGATTCTAAGGTTCCAATTATCATGATTGGTCCAGG
TACTGGTTTGGCTCCTTTTAGAGGTTTTTTACAAGAAAGATTGGCCTTGGTCGAATCCG
GTGTTGAATTGGGTCCATCTGTTTTGTTTTTCGGTTGCAGAAACAGAAGAATGGACTTC
ATCTACGAAGAAGAATTACAAAGATTCGTCGAATCAGGTGCTTTGGCAGAATTGTCAG
TTGCTTTTTCTAGAGAAGGTCCAACAAAAGAATACGTCCAACACAAGATGATGGATAA
GGCTTCTGATATCTGGAACATGATTTCTCAAGGTGCCTACTTGTATGTTTGTGGTGATG
CTAAAGGTATGGCCAGAGATGTTCATAGATCCTTGCATACAATTGCCCAAGAACAAGG
TTCTATGGACTCTACAAAAGCAGAAGGTTTCGTCAAGAACTTGCAAACTTCTGGTAGAT
ACTTGAGAGATGTCTGGTGA

DNA sequence of *At4CL2* codon-optimized for *S. cerevisiae* by GeneArt (Life Technologies)

ATGACTACCCAAGATGTTATCGTCAACGATCAAAACGACCAAAAGCAATGTTCCAACG
ATGTCATCTTCAGATCTAGATTGCCAGATATCTACATCCCAAACCATTTGCCATTGCAC
GATTACATCTTCGAAAACATTTCTGAATTCGCTGCTAAGCCATGCTTGATTAACGGTCC
AACTGGTGAAGTTTACACTTACGCTGATGTTCATGTTACCTCTAGAAAATTGGCTGCTG
GTTTACACAATTTGGGTGTTAAGCAACACGATGTCGTTATGATTTTGTTGCCAAACTCT
CCAGAAGTTGTCTTGACTTTTTTGGCTGCTTCTTTCATTGGTGCTATTACTACTTCTGCT
AACCCATTTTTTACCCCAGCCGAAATTTCTAAACAAGCTAAAGCTTCTGCTGCCAAGTT
GATCGTTACTCAATCAAGATACGTTGACAAGATCAAGAACTTGCAAAACGATGGTGTT
TTGATTGTCACCACTGATTCTGATGCTATTCCAGAAAACTGCTTGAGATTCTCTGAATT
GACCCAATCTGAAGAACCTAGAGTTGATTCCATCCCAGAAAAGATTTCACCAGAAGAT
GTTGTTGCTTTGCCATTCTCTTCAGGTACTACTGGTTTGCCAAAAGGTGTTATGTTGACT
CATAAGGGTTTGGTTACATCCGTTGCTCAACAAGTTGATGGTGAAAATCCAAACTTGTA
CTTCAACAGAGATGACGTTATCTTGTGCGTTTTGCCAATGTTTCATATCTACGCCTTGA
ACTCCATCATGTTGTGTTCTTTGAGAGTTGGTGCCACCATTTTGATTATGCCAAAGTTCG
AAATCACCTTGTTGTTGGAACAAATCCAAAGATGCAAGGTTACCGTTGCTATGGTTGTT
CCACCAATAGTTTTGGCTATTGCTAAGTCTCCAGAAACCGAAAAGTACGATTTGTCCTC
TGTTAGAATGGTTAAGTCTGGTGCTGCTCCATTGGGTAAAGAATTGGAAGATGCTATTT
CTGCTAAGTTCCCAAATGCTAAGTTGGGTCAAGGTTATGGTATGACTGAAGCTGGTCCA
GTTTTAGCTATGTCTTTGGGTTTTGCTAAAGAACCATTCCCAGTAAAATCTGGTGCTTGT
GGTACTGTTGTTAGAAACGCTGAAATGAAGATTTTGGACCCAGATACTGGTGATTCTTT
GCCAAGAAACAAACCAGGTGAAATATGCATCAGAGGTAATCAAATCATGAAGGGTTA
CTTGAACGATCCATTGGCTACTGCTTCTACCATTGATAAGGATGGTTGGTTGCATACAG
GTGATGTTGGTTTCATAGATGATGACGACGAATTATTCATCGTTGATAGATTGAAAGAA
TTGATCAAGTACAAGGGTTTCCAAGTTGCTCCAGCTGAATTGGAATCTTTGTTGATTGG
TCATCCAGAAATCAACGACGTTGCTGTTGTTGCAATGAAGGAAGAAGATGCCGGTGAA
GTTCCAGTTGCTTTCGTTGTTAGATCCAAGGATTCTAACATCTCCGAAGACGAAATCAA
GCAATTCGTTTCTAAGCAAGTCGTTTTCTACAAGAGAATCAACAAGGTTTTCTTCACCG
ACTCTATTCCAAAAGCTCCATCTGGTAAGATCTTGAGAAAGGATTTGAGAGCTAGATT
GGCTAACGGTTTGATGAACTGA

DNA sequence of *VvVST1* codon-optimized for *S. cerevisiae* by GeneArt (Life Technologies)

ATGGCTTCCGTTGAAGAATTCAGAAACGCTCAAAGAGCTAAAGGTCCAGCTACTATTT
TGGCTATTGGTACTGCTACTCCAGATCATTGTGTTTACCAATCTGATTACGCCGACTAC
TACTTCAGAGTTACTAAGTCTGAACACATGACCGAATTGAAGAAAAAGTTCAACAGAA
TCTGCGACAAGTCCATGATCAAGAAGAGATATATCCACTTGACCGAAGAAATGTTGGA
AGAACATCCAAACATTGGTGCTTATATGGCTCCATCCTTGAACATCAGACAAGAAATT
ATCACTGCCGAAGTTCCAAGATTGGGTAGAGATGCTGCTTTGAAGGCTTTGAAAGAAT
GGGGTCAACCTAAGTCTAAGATCACCCATTTGGTTTTCTGTACTACCTCTGGTGTTGAA
ATGCCAGGTGCTGATTACAAATTGGCTAACTTGTTGGGTTTGGAAACCTCCGTTAGAAG
AGTTATGTTGTACCATCAAGGTTGTTATGCTGGTGGTACTGTTTTGAGAACTGCTAAAG
ATTTGGCTGAAAACAATGCTGGTGCTAGAGTTTTGGTTGTTTGCTCTGAAATTACCGTT
GTTACTTTCAGAGGTCCATCTGAAGATGCTTTGGATTCTTTGGTTGGTCAAGCTTTGTTT
GGTGATGGTTCTTCTGCTGTTATAGTTGGTTCTGATCCAGATGTCTCTATCGAAAGACC
TTTGTTCCAATTGGTTTCTGCTGCTCAAACTTTCATTCCAAATTCTGCTGGTGCAATTGC
TGGTAACTTGAGAGAAGTTGGTTTGACTTTTCATTTGTGGCCAAACGTTCCAACTTTGA
TCTCCGAAAACATTGAAAAGTGTTTGACCCAAGCTTTCGATCCATTGGGTATTTCTGAT
TGGAATTCCTTGTTCTGGATTGCTCATCCAGGTGGTCCAGCAATTTTGGATGCTGTTGA
AGCTAAATTGAACTTGGAAAAGAAGAAGTTGGAAGCCACCAGACATGTTTTGTCTGAA
TACGGTAATATGTCCTCTGCTTGCGTTTTGTTCATTTTGGACGAAATGAGAAAAAAGTC
CTTGAAGGGTGAAAAGGCTACTACTGGTGAAGGTTTGGATTGGGGTGTTTTGTTCGGTT
TTGGTCCAGGTTTGACTATTGAAACTGTTGTCTTGCATTCTGTTCCAACCGTTACCAATT
GA

DNA sequence of *SbROMT* codon-optimized for *S. cerevisiae* by GeneArt (Life Technologies)

ATGGTCTTGATCTCCGAAGATTCCAGAGAATTATTGCAAGCCCATGTCGAATTGTGGAA
TCAAACTTACTCTTTCATGAAGTCCGTTGCTTTGGCTGTTGCTTTAGACTTGCATATTGC
TGATGCCATTCATAGAAGAGGTGGTGCTGCTACTTTGTCTCAAATTTTGGGTGAAATTG
GTGTCAGACCATGTAAATTGCCAGGTTTACACAGAATCATGAGAGTCTTGACTGTTTCT
GGTACTTTCACTATCGTTCAACCATCTGCTGAAACCATGTCATCTGAATCTGATGGTAG
AGAACCAGTTTACAAGTTGACTACTGCTTCCTCTTTGTTGGTTTCCTCTGAATCTTCTGC
TACAGCTTCTTTGTCTCCAATGTTGAACCATGTTTTGTCCCCATTCAGAGATTCTCCATT
GTCTATGGGTTTGACTGCTTGGTTTAGACACGATGAAGATGAACAAGCTCCAGGTATGT
GTCCTTTTACTTTGATGTATGGTACTACCTTGTGGGAAGTCTGTAGAAGAGATGATGCT
ATTAACGCCTTGTTCAACAATGCTATGGCTGCTGATTCTAACTTCTTGATGCAAATCTT
GTTGAAAGAATTCTCCGAAGTTTTCTTGGGTATCGACTCTTTGGTTGATGTTGCTGGTG
GTGTTGGTGGTGCTACTATGGCTATTGCTGCTGCTTTTCCATGTTTGAAGTGTACCGTTT
TGGATTTGCCACATGTTGTTGCTAAAGCTCCATCTTCTTCTATCGGTAACGTTCAATTTG
TCGGTGGTGATATGTTCGAATCTATTCCACCAGCTAACGTCGTTTTGTTGAAATGGATT
TTACACGACTGGTCCAACGATGAATGCATTAAGATTTTGAAGAACTGCAAGCAAGCCA
TCCCATCTAGAGATGCCGGTGGTAAGATTATTATCATCGATGTTGTTGTCGGTTCCGAT
TCTTCTGATACAAAGTTGTTGGAAACCCAAGTCATCTACGACTTGCATTTGATGAAGAT
TGGTGGTGTCGAAAGAGATGAACAAGAATGGAAGAAGATTTTCTTGGAAGCCGGTTTC
AAGGACTACAAGATTATGCCAATTTTAGGTTTGAGATCCATCATCGAATTATACCCATG
A

DNA sequence of *VvROMT* codon-optimized for *S. cerevisiae* by GeneArt (Life Technologies)

ATGGATTTGGCCAACGGTGTTATTTCCGCTGAATTATTGCATGCTCAAGCTCATGTTTG
GAACCACATTTTCAACTTCATCAAGTCCATGTCTTTGAAGTGCGCTATTCAATTGGGTA
TCCCAGATATCATTCATAACCATGGTAAGCCAATGACCTTGCCAGAATTGGTTGCTAAA
TTGCCAGTTCACCCAAAAAGATCTCAATGCGTTTACAGATTGATGAGAATCTTGGTCCA
TTCTGGTTTTTTGGCTGCTCAAAGAGTTCAACAAGGTAAAGAAGAAGGTTACGTTT
TGACCGATGCCTCTAGATTGTTGTTGATGGATGATTCCTTGTCCATCAGACCATTGGTTT
TGGCTATGTTGGATCCTATTTTGACTAAGCCATGGCATTATTTGTCCGCCTGGTTTCAAA
ATGATGACCCAACTCCATTTCATACCGCTCACGAAAGATCATTTTGGGATTATGCTGGT
CATGAACCACAATTGAACAACTCATTCAATGAAGCTATGGCTTCCGATGCTAGATTATT
GACTTCCGTCTTGTTGAAAGAAGGTCAAGGTGTTTTTGCTGGTTTGAACTCATTGGTTG
ATGTTGGTGGTGGTACTGGTAAAGTTGCTAAAGCTATTGCTAATGCCTTCCCACATTTG
AACTGTACCGTTTTGGATTTGCCACATGTTGTTGCAGGTTTACAAGGTTCTAAGAACTT
GAATTACTTCGCCGGTGATATGTTCGAAGCTATTCCACCAGCTGATGCTATTTTGTTGA
AATGGATATTGCACGACTGGTCCGATGAAGAATGTGTTAAGATTTTGAAGAGATGCAG
AGAAGCCATCCCATCTAAAGAAAATGGTGGTAAGGTTATCATCATCGACATGATTATG
ATGAAGAATCAAGGTGACTACAAGTCCACTGAAACCCAATTATTCTTCGACATGACCA
TGATGATTTTCGCTCCAGGTAGAGAAAGAGATGAAAACGAATGGGAAAAGTTGTTCTT
GGATGCTGGTTTCTCCCATTACAAGATTACTCCAATTTTGGGTTTGAGATCCTTGATCG
AAGTTTACCCATGA

References

1. **Jensen NB, Strucko T, Kildegaard KR, David F, Maury J, Mortensen UH, Forster J, Nielsen J, Borodina I.** 2014. EasyClone: method for iterative chromosomal integration of multiple genes in *Saccharomyces cerevisiae*. FEMS Yeast Res **14:**238-248.
2. **Norholm MH.** 2010. A mutant Pfu DNA polymerase designed for advanced uracil-excision DNA engineering. BMC Biotechnol **10:**21.
3. **Gietz RD, Woods RA.** 2002. Transformation of yeast by lithium acetate/single-stranded carrier DNA/polyethylene glycol method. Methods Enzymol **350:**87-96.
4. **Li M, Kildegaard KR, Chen Y, Rodriguez A, Borodina I, Nielsen J.** 2015. *De novo* production of resveratrol from glucose or ethanol by engineered *Saccharomyces cerevisiae*. Metab Eng **32:**1-11.
5. **Stovicek V, Borodina I, Forster J.** 2015. CRISPR–Cas system enables fast and simple genome editing of industrial *Saccharomyces cerevisiae* strains. Metabolic Engineering Communications **2:**13-22.
6. **Rodriguez A, Kildegaard KR, Li M, Borodina I, Nielsen J.** 2015. Establishment of a yeast platform strain for production of *p*-coumaric acid through metabolic engineering of aromatic amino acid biosynthesis. Metabolic engineering **31:**181-188.
7. **Maury J, Germann SM, Baallal Jacobsen SA, Jensen NB, Kildegaard KR, Herrgard MJ, Schneider K, Koza A, Forster J, Nielsen J, Borodina I.** 2016. EasyCloneMulti: A Set of Vectors for Simultaneous and Multiple Genomic Integrations in *Saccharomyces cerevisiae*. PLoS One **11:**e0150394.
8. **Ronda C, Maury J, Jakociunas T, Jacobsen SA, Germann SM, Harrison SJ, Borodina I, Keasling JD, Jensen MK, Nielsen AT.** 2015. CrEdit: CRISPR mediated multi-loci gene integration in *Saccharomyces cerevisiae*. Microb Cell Fact **14:**97.
